# Headspace Volatile Organic Compound (VOC) Profiling of Infected and Non-Infected Wound Swabs—A Pilot Study

**DOI:** 10.3390/biom16050634

**Published:** 2026-04-24

**Authors:** Shane Fitzgerald, Linda Holland, Melissa Finnegan, Kellie Fortune, Brid Cooney, Eoghan O’Neill, John H. McDermott, Seamus Sreenan, Tommy Kyaw-Tun, Aoife Morrin

**Affiliations:** 1 School of Chemical Sciences, Insight Research Ireland Centre for Data Analytics, Dublin City University, D09 V209 Dublin, Ireland; melissafinnegan33@gmail.com; 2 School of Biotechnology, Dublin City University, D09 V209 Dublin, Ireland; linda.holland@dcu.ie; 3 Academic Department of Endocrinology and Diabetes, Connolly Hospital, Royal College of Surgeons in Ireland, D15 X40D Dublin, Ireland; kellie.fortune@hse.ie (K.F.); brid.cooney@hse.ie (B.C.); johnmcdermott@rcsi.ie (J.H.M.); ssreenan@rcsi.ie (S.S.); tommykyawtun@rcsi.ie (T.K.-T.); 4 Department of Clinical Microbiology, RCSI University of Medicine and Health Sciences, RCSI Education & Research Centre, D09 YD60 Dublin, Ireland; eoneill@rcsi.com; 5 Department of Microbiology, Connolly Hospital, D15 X40D Dublin, Ireland; 6 Health Research Board Diabetes Collaborative Clinical Trial Network, H91 V4AY Galway, Ireland

**Keywords:** wound, infection, VOCs, GCMS, SPME

## Abstract

Infections of chronic wounds are a major healthcare burden worldwide and can lead to poor health outcomes such as amputations of limbs and death. Detecting infections early significantly increases the effectiveness of therapeutic interventions. Screening of volatile organic compounds (VOCs) emitted from wound swab samples can potentially serve as a highly specific indicator of infection. Profiling of VOCs from infected and non-infected wounds was carried out. Swab samples were collected from 26 wounds from 23 patients (n = 20 diabetic patients; n = 3 non-diabetic patients). There were 16 wounds sampled that were clinically determined as infected, and 10 as non-infected. Headspace-solid phase microextraction gas chromatography-mass spectrometry (HS-GC-MS) was used to rapidly sample and detect VOCs from the swabs following a short incubation period. A total of 42 compounds were identified and included for analysis. Infected wounds emitted more diverse VOCs compared to non-infected wounds. Higher numbers of compounds with significantly higher abundances were detected from severely infected wounds compared to less severely infected wounds. Abundances of short-chain fatty acids (SCFAs) and branched-chain fatty acids (BCFAs) were found to be the strongest discriminators of infected from non-infected wounds. Further validation is needed, but the results of this pilot study highlight the potential of detecting these compounds as a highly specific and targeted route to predicting or detecting wound infections in the future.

## 1. Introduction

Chronic wounds are characterised by the impairment and prolonging of the healing process. Diabetic foot ulcers (DFUs) are a significant contributor to the chronic wound incidence rate worldwide. Ulceration is typically caused by abnormal pressure applied to the foot—which diabetic ischaemia renders the skin less able to withstand—resulting in a break in the skin [1]. It is estimated that around one in four people with diabetes will develop a chronic DFU in their lifetime [2]. Infections of chronic wounds are powerful risk factors for emergency department visits and hospital admissions [2]. Longitudinal observational studies monitoring hundreds of patients with DFUs [3] have highlighted the persistence and ultimate danger that they pose, as lower extremity amputations, revascularisation and death are often the clinical outcomes that occur as a result of these wounds. Chronic wounds can also occur from venous ulcers, post-surgical complications, and injuries sustained on battlefields.

Early detection of wound infections is a highly effective therapeutic intervention [4,5]. However, accurately diagnosing an infection is currently limited by challenges associated with characterising the presence and severity of an infection, and/or differentiating soft tissue from osteomyelitis (bone infections) [6]. Clinically, signs such as erythema, pus formation and odour may help guide diagnoses. Inflammatory blood biomarkers such as white blood cell (WBC) count, erythrocyte sedimentation rate (ESR), C-reactive protein (CRP) and procalcitonin (PCT) are also measured to aid the diagnosis of infection. These markers are non-specific and are upregulated in response to inflammation and, therefore, are influenced by many other processes and co-morbidities. Blood marker levels are also influenced by anti-inflammatory medication and antibiotics [7], which can complicate accurate diagnoses. Although culture-based methods are widely considered standard protocol for detecting bacteria in wounds, the turnaround time of results is significant at 36–48 h [4]. Volatile organic compounds (VOCs) being emitted from wounds have the potential to provide targeted chemical and metabolic information that may support clinical judgement of the infection state [4,8].

VOC profiling has been used for characterising small metabolites and potential biomarkers of microbial pathogens and various diseases. Biological VOCs are low molecular weight molecules generated as end products from a variety of metabolic mechanisms in living organisms, and several reviews [9,10] and books [11] provide comprehensive references for microbial and clinical volatilomics. Resulting developments in this area could potentially give rise to rapid diagnostic and monitoring capabilities for a variety of diseases and infections [12]. Disease-associated volatilomic shifts have been reported for various diseases and maladies, including tuberculosis [13] and bacterial lung infection [14]. Volatilomic wound profiling methodology was first applied to discriminate chronic wound emissions from healthy skin controls [15] but without consideration of the clinical judgement of infection. While malodour is commonly associated with wound infection and has been attributed to bacterial metabolites, dimethyl disulfide [16] and dimethyl trisulfide [17,18], it is hypothesised that a comprehensive profiling of VOCs from wound tissue or swabs could be more robust as a diagnostic tool to support clinical judgement. Clear discrimination of VOC profiles of non-infected and infected wound swabs was demonstrated using e-nose technology [19]. Another study described significant VOC profile discrimination between non-infected and lab-infected wound tissue biopsies using headspace sampling with GCMS analysis [20]. Both papers highlight the clinical potential of rapid discrimination of wound infections using VOC profiling.

In this study, volatilomic profiling of wound swabs was undertaken for the first time to investigate its potential in determining wound infection status. The HS-SPME-GC-MS analytical workflow allowed the detection of diverse and broad volatile profiles within 3 h after sample collection. The primary aim of this work was to characterise the VOCs detected across infected and non-infected wound swabs and to assess the presence of any infection-specific compound emissions. Volatilomic differences across clinically determined infection severities were investigated at the compound level. The predictive ability of these compounds to detect clinical infection was assessed. A panel of fatty acids (FAs)—comprising short-chain FAs (SCFAs) and branched-chain FAs (BCFAs)—was investigated for the first time for their ability to distinguish between non-infected wounds and those that require clinical intervention, highlighting the potential of volatilomics as a diagnostic support in the clinical assessment of wound infection.

## 2. Materials and Methods

### 2.1. Participant Profile

Twenty-three patients with chronic wounds attending Connolly Hospital (Blanchardstown, Dublin) were recruited. Of these, twenty patients had diabetes (Type 2: n = 19; Type 1: n = 1) and were being treated in the podiatry department of the Diabetes Day Centre within the hospital; three patients (Nondiabetic) were recruited from non-diabetic out-patient care (Table S1). All participants’ personal data were anonymised in accordance with GDPR guidelines. Participants were asked to provide written informed consent on the day of sampling. The study was approved by the Research Ethics Committees of Dublin City University and Connolly Hospital Research. The study was performed as fully blinded to the GC-MS analyst until all volatile analysis and data processing were complete.

All wounds were clinically assessed, and infection state (Infected/Non-infected) determined by a consultant diabetologist (TKT) and clinical podiatry specialist (KF) based on the history, physical examination, blood tests (C-reactive protein (CRP), white blood cell (WBC) count, neutrophils (Neut), microbiological plating results (with input of the consultant microbiologist (EON)), and x-rays or magnetic resonance imaging in cases of suspected osteomyelitis. Other clinical tests, such as the probe-to-bone test, were also used to assess wound depth and indicate potential osteomyelitis. Physical infection cues that were noted during clinical appointments included discolouration, oedema, pus formation, and malodour. Infection state was diagnosed as accurately as allowable using available methods in line with the guidelines outlined by the International Working Group of the Diabetic Foot (IWGDF) [5]. The clinical tests, along with visual examination, allowed the clinical team to determine a Texas score and infection state to categorise each diabetic wound and infection state only for nondiabetic wounds (Table S2).

### 2.2. Wound Swab Collection and VOC Sampling

Following consent, patient wounds were exposed, cleansed with saline and debrided where clinically indicated. Wound infection status was classified with a Texas score, based on the University of Texas classification system (Table S2) [21] and used by clinicians to characterise the depth and infection state of DFUs. All swabs were taken using a zigzag method [22]. Three swab samples were taken from the wound using standard clinical wound swabs. One sample was sent to the local microbiology laboratory for routine culture and sensitivity. The remaining two were individually sealed in media-free glass 20 mL headspace (HS) (Sigma-Aldrich, Gillingham, UK) vials and transported to Dublin City University for duplicate GC-MS analysis. Glass vials were heated to 37 °C for 2 h to allow the compounds present on the swab substrate to reach equilibrium with the glass HS.

### 2.3. Gas Chromatography–Mass Spectrometry (GCMS) Analysis

An Agilent 6890 GC connected to an Agilent 5973 mass selective detector (Agilent Technologies, Inc., Santa Clara, CA, USA) was used for all compound analyses. Separations were performed on a DB-WAX column (Agilent Technologies Ireland, Cork, Ireland) (30 m × 0.25 mm × 0.32 μm). The carrier gas used was helium, with a constant flow rate of 1.3 mL/min. For manual injections of SPME fibres, the system was equipped with a SPME Merlin Microseal (Merlin Instrument Company, Newark, DE, USA), and the inlet was maintained at a temperature of 280 °C. Split-less injection was used for all samples, with a gas purge (50 mL/min) being activated after 2 min. Prior to HS sampling, SPME fibres (Merck KGaA, Darmstadt, Germany) were conditioned in the GC thermal desorption inlet at 280 °C. After 2 h incubation, a SPME fibre (Carboxen/PDMS) was exposed to the sample HS for 20 min. After 20 min, the fibre was retracted and transferred to the injector port of the GC for desorption. The SPME fibre was desorbed for 2 min at 280 °C within the inlet liner. The initial GC oven temperature was 40 °C for 5 min and was programmed to increase at a rate of 10 °C min^−1^ to 240 °C, with a final hold for 5 min at this temperature, giving an overall running time of 29 min. The transfer line temperature was set at 230 °C. The MS was operated at a scan range of 35–400 *m*/*z*, scan rate of 3.94 s^−1^, ion source temperature 230 °C and ionising energy of 70 eV.

### 2.4. Feature Extraction, Peak Identification and Data Analysis

Identification of compounds was performed using the National Institute of Standards and Technology (NIST) library (2017)—match factors > 70% were used. Compounds that showed significant correlations with infection status were more typically matched to >85% across wound samples analysed. Only chromatographic peaks with a signal-to-noise (S/N) ratio greater than 3:1 were considered for identification. Retention index (RI) values for polar columns provided by the NIST Chemistry WebBook were used to support compound identification. Any compound found to have an RI value ≤ 12 RI units of the RI values in the NIST database was deemed acceptable as a match. A standard mixture of saturated alkanes in hexane (C_7_–C_30_; Merck, Cork, Ireland) was run under the same temperature conditions as the samples and used for RI matching. This was done by rapidly dipping an already exhausted SPME needle into the mixture once and injecting it into the GC-MS. A fully functional SPME fibre was not used for this because exposure to hexane degrades the fibre integrity. The identification of acetic acid (CAS: 64-19-7), 3-methylbutanoic acid (CAS: 503-74-2); 2-methylbutanoic acid (CAS: 116-53-0), butanoic acid (CAS: 107-92-6), propanoic acid (CAS: 79-09-4), 2-methylpropanoic acid (CAS: 79-31-2), ethyl acetate (CAS: 141-78-6) and phenylethyl alcohol (CAS: 60-12-8, CAT code: 1533250) were validated by standards injection. All standards were obtained from Merck. Relative ion abundance data for each pure standard were recorded and manually used to aid the identification of these compounds in clinical samples.

### 2.5. Data Analysis

Raw VOC data was standardised using centering and scaling [23]. Compounds that were present in some samples and absent from others were input as zero when absent. R (version: 2023.06.0+421). packages used for the graphics in this study were: ‘tidyverse’ (version: 1.3.1), ‘ggplot2’ (version: 3.3.5), ‘ggfortify’ (version: 0.4.12).

### 2.6. Method Development

#### 2.6.1. Indoor Air and Blank Swab Sampling

Indoor air and blank swabs were sampled and analysed periodically to establish background volatile profiles over the course of the study, and potential exogenous compounds were identified (Table S3). Chromatographic peaks corresponding to these identified compounds in wound swab samples with signal-to-noise ratios < 3:1 were considered background and not included in the final dataset.

#### 2.6.2. Swab Sample Stability

The forearms of 2 healthy participants (1 male, 1 female) were swabbed (~5 s) by rotating a swab over a defined 1 cm^2^ area of the skin, applying pressure, and the samples were sealed in HS vials. The vials containing the swabs were left at room temperature for 1 h to allow for the time period in the study between sample collection and transport to the laboratory for analysis. To assess the effect of elapsed time between sample collection and analysis on the VOC profiles, samples were then incubated at 37 °C for 2, 4, 23 and 47 h before being sampled with SPME and analysed using GC-MS (n = 3).

#### 2.6.3. Reproducibility

The forearms of 5 healthy participants (3 male, 2 female) were swabbed in triplicate. Following the described sampling and analysis procedure, the detection and peak areas of selected skin-associated VOCs were determined across replicate samples of each participant. Reproducibility was characterised by the average %RSD across the 5 participants.

#### 2.6.4. Discriminatory Power of Headspace Analysis of Swab Samples

On a single participant, the forearm, abdomen and axillae (armpit) were swabbed in triplicate and the samples sealed in HS vials. Following 2 h of incubation at 37 °C, the swabs were sampled and analysed using HS-SPME-GCMS, and the VOC profiles recovered were compared.

## 3. Results

### 3.1. Method Validation

Swab sample VOC stability was assessed by comparing the influence of different waiting periods between sample collection (skin) and analysis. Figure S1 shows the abundances of 2 skin VOCs (nonanal and 5-hepten-2-one, 6-methyl-) after 3, 5, 24, and 48 h incubation post sample collection. The decline in VOC abundances recovered after 3 h highlights the preference for shorter incubation for optimum analysis signal post-sample-collection. As a result, 3 h was chosen as the incubation period for the wound samples.

Five participants were swabbed in triplicate, and skin-associated VOCs were identified [24] and %RSDs of selected peak areas were used to determine the relative reproducibility of the swab-HS-SPME-GCMS method (Table S4). The capability of swab sampling for VOC profiling and discrimination was also assessed by analysing swab samples taken from different points of the body from a single healthy male participant (Figure S1). The heatmap shown in Figure S2 visualises clear discrimination of VOC profiles of forearm, abdomen and axillae (armpit) triplicate swab samples.

### 3.2. Infected Wounds Show More Chemically Diverse Volatilomes than Non-Infected Wounds

**Figure 1:**
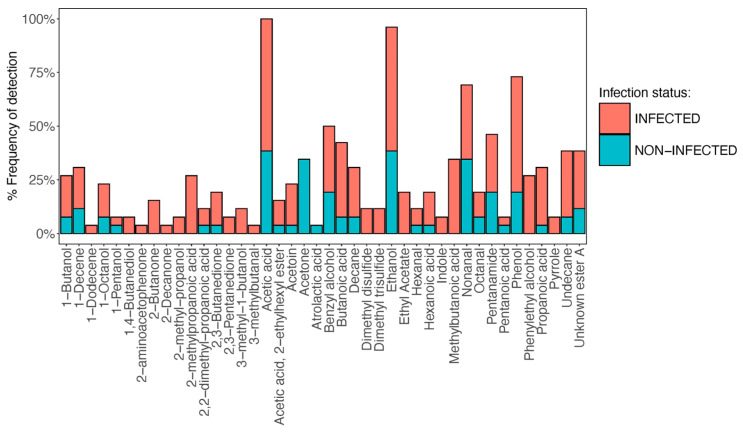
Frequency bar plot showing % of samples in which each VOC was detected in infected and non-infected wound samples. The total number of wound samples clinically determined to be infected was 16, and the number of non-infected samples was 10.

**Figure 2:**
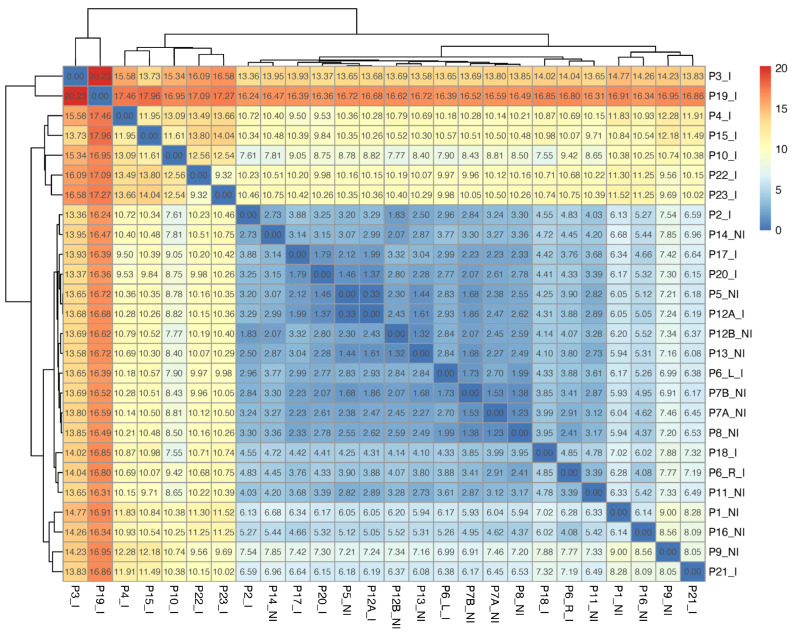
Dissimilarity matrix—Dissimilarity of sample VOC profiles is calculated by Euclidean distance. The numbers present on each cell represent the Euclidean distance of sample (row) to sample (column).

Following GC-MS analysis and background subtraction, a total of 42 compounds were identified across the wound samples and included in this study. Detection frequencies for individual compounds are shown in Figure 1. Frequently detected compounds across both infected and non-infected samples included acetic acid, acetone and ethanol. Low abundances of these compounds were also detected at an S/N ratio < 3:1 in various blank swab samples. Higher numbers of compounds were detected in infected wound swabs (Figure S1). Clinical information for all wounds analysed is present in Table S1. The dissimilarity matrix shown in Figure 2 broadly visualises the differences and similarities in whole wound VOC profiles across the sample cohort. Swabs collected from DFUs with a deep bone infection (osteomyelitis, Texas score 3B—wounds P4, P10, P15, P19 (Table S1)) had markedly dissimilar VOC profiles from those of less severely infected and non-infected wounds. The VOC profile of P3 was also highly dissimilar; this was a non-diabetic wound that was diagnosed with a chronic mixed anaerobic infection. Although VOC profiles of less severely infected wounds could be discriminated from non-infected wounds at the compound-level, profile-level differences were not visualised due to the significantly higher dissimilarity of severely infected wounds (Figure 2). The compounds detected across the cohort with respect to recorded Texas scores are shown in Figures S4–S8. SCFAs butanoic and propanoic acids, as well as BCFAs 2-methylpropanoic acid and methylbutanoic acid, were detected in greater abundances and frequencies from infected wound samples (Figure 1). In the cases of wounds in which methylbutanoic acid was detected, 60 *m*/*z* was the dominant ion in the mass spectrum of the chromatographic peak, indicating higher abundances of the 3-methylbutanoic acid isomer (base ion: 60 *m*/*z*). However, notable abundances of 74 *m*/*z* ion were also detected (Figure S3), indicating lower amounts of the 2-methylbutanoic acid isomer (base ion: 74 *m*/*z*) were also present. Phenylethyl alcohol was also highly prevalent in infected samples. Other compounds, such as acetoin, 2-butanone, dimethyl disulfide, dimethyl trisulfide, 3-methyl-1-butanol, 2-methyl-1-propanol, indole, and ethyl acetate, were detected in lower frequencies but had high specificity in infected wound samples. Compounds consistently detected across all samples included nonanal, octanal, ethanol, acetone, benzyl alcohol and phenol.

### 3.3. Differences in Abundance of Key Compounds Between Infected and Non-Infected Wounds

**Figure 3:**
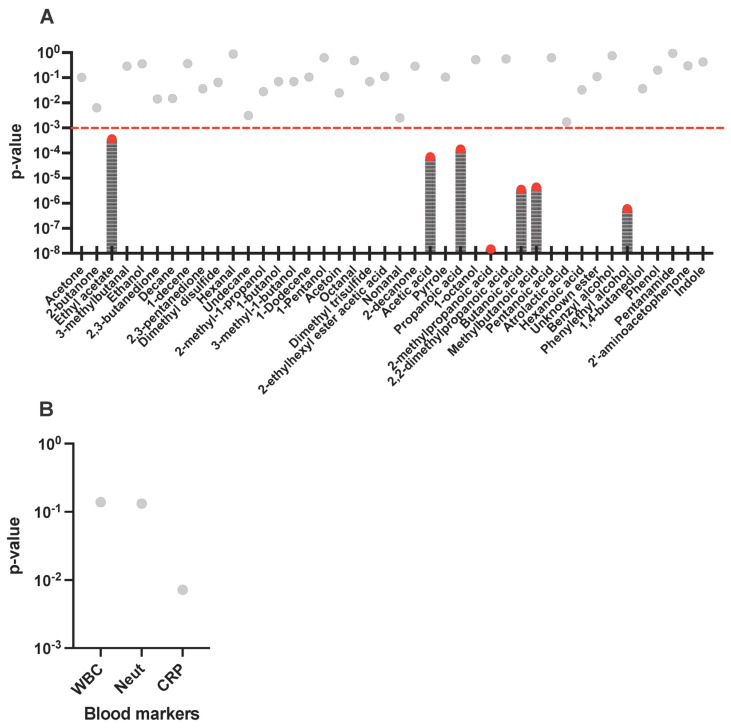
*p*-values for Spearman correlation coefficients with infection status from 26 wound swab samples (infected: n = 16; non-infected: n = 10) (**A**) VOCs and (**B**) blood markers. *p*-value < 0.001 (dashed red line) and marked as red dots were deemed significant. Grey vertical lines for visualisation only. WBC: white blood cell count; Neut: Neutrophils; CRP: C-reactive protein (CRP).

**Figure 4:**
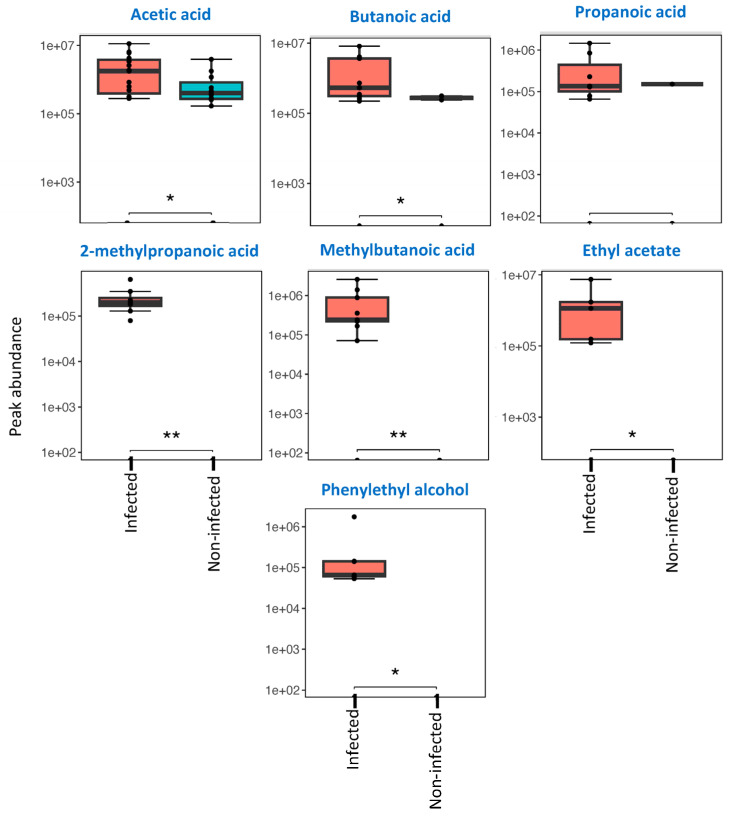
Comparative boxplots assessing differences in abundances of compounds that had strong correlations with infection status across the 26 wound samples analysed (infected: n = 16; non-infected: n = 10). Due to the non-parametric nature of the data, the Wilcoxon rank sum test was used to assess the strength of differences. Differences are illustrated through the star system, where the number of * indicates higher discrimination. The following symbols were used to indicate statistical significance (ns: *p* > 0.05; *: *p* ≤ 0.05; **: *p* ≤ 0.01).

Spearman correlation coefficients were calculated to assess the association of each compound with infection status. Asymptotic *p*-values were calculated to determine the approximate significance of the compound-infection state Spearman coefficient values. Following this, 7 compounds were determined to have the strongest correlation with either infection or absence of infection (*p*-value < 0.001); these are shown in Figure 3A. These were acetic acid, butanoic acid, propanoic acid, methylbutanoic acid, 2-methylpropanoic acid, ethyl acetate, and phenylethyl alcohol. Blood marker abundances were also subjected to this analysis (Figure 3B), demonstrating that WBC count and Neut showed no correlation with infection. CRP showed the strongest correlation with infection in this study (*p*-value = 0.0072). Univariate analysis was used to assess specific compound-level differences in abundance between infected and non-infected groups (Figure 4). Figure 4 compares the median abundances for the discriminating 7 compounds (Figure 3A) across infected and non-infected groups. The Wilcoxon rank sum test was employed to determine significance in differences between infected and non-infected groups. Out of the 7 compounds, significant differences were observed in 6 compounds, 2 of which were SCFAs (acetic acid, butanoic acid); 2 were BCFAs (2-methylpropanoic acid and methylbutanoic acid); and also phenylethyl alcohol and ethyl acetate. Compound abundance differences according to Texas score for the samples taken from DFUs are available in Figures S4–S8, which visualise the abundance distribution for each compound and chemical class across the various Texas scores registered for the DFUs in this study. The highest abundances and diversity of compounds were observed in samples collected from patients with osteomyelitis (Texas score 3B). Relatively low abundances of acetic acid were detected across all non-infected samples and in blank swab samples.

### 3.4. Fatty Acids Indicators of Potential Infections in Wounds

**Figure 5:**
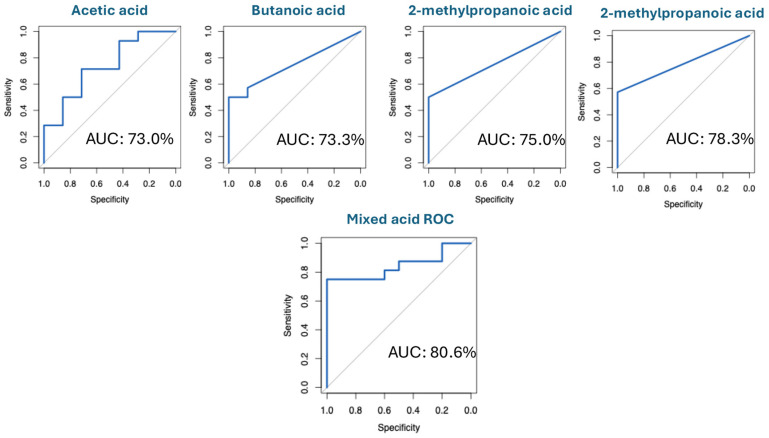
ROC curves for individual FAs (**top**) were constructed to determine the predictive power of each compound for infection; this is described by the AUC (%). The mixed acid ROC model (**bottom**) was constructed by combining the individual models of acetic acid, butanoic acid, 2-methylpropanoic acid and methylbutanoic acid. These plots were generated using the 26 wound samples analysed (infected: n = 16; non-infected: n = 10).

The six highly discriminating compounds shown in Figure 4 were subsequently used as variables in regression analysis to assess the relationship between their detection and infection status. To assess the predictive power of selected compounds for infection, logistical regression analysis was used to generate ROC curves for compounds that had a strong correlation with infection. Logistic regression was used for this analysis as outcomes (infected and non-infected) are binary. Individual ROC curves for each of the four FAs (SCFAs and BCFAs) were constructed and are shown in Figure 5. Sensitivity (%) of the analysis was determined by the true positive rate (%), while the specificity (%) of the analysis is determined by 100%—false positive rate (%); the lower the false positive rate (%), the higher the specificity (%). The performance (accuracy) of the model is represented by the area-under-the-curve percentage (AUC%). To reduce the risk of overfitting, we performed 5-fold cross-validation. In each iteration, the model was trained on 80% of the data and evaluated on the remaining 10%. Cross-validated AUC values were averaged across folds to provide an unbiased estimate of predictive performance. Methylbutanoic acid was the strongest predictor of infection, with a sensitivity of 56.2% and a specificity of 100%. Acetic acid, butanoic acid and 2-methylpropanoic acid also performed relatively well with sensitivities of 100%, 56.2% and 50.0% and specificities of 0%, 80% and 100%, respectively. To assess whether grouping the regression improved predictive performance, multiple regression analysis was carried out. By grouping the regressions of acetic acid, butanoic acid, 2-methylpropanoic acid and methylbutanoic acid collectively against infection status, the prediction model performance increased to 80.6%.

## 4. Discussion

In this study, a novel HS-SPME GCMS analysis workflow was employed to investigate the potential application of volatile chemistry for the rapid clinical assessment of wound infections. The primary aim of this study was to characterise and investigate compound-level differences in the volatilome of 26 wound swab samples taken from infected (n = 16) and non-infected (n = 10) using HS-SPME-GC-MS. Swabbing is a rapid and internationally standardised method of non-invasively collecting a clinical sample. Although culture-based methods are widely considered standard protocol for detecting bacteria in wounds, the turnaround time of results is significant at 36–48 h [4]. The turnaround time of data from this VOC method was optimised to 3.5 h post-sample collection. As mass spectrometry is taking on a growing role in modern diagnostic role in modern hospitals [25], turnaround times could be further reduced by on-site GCMS capabilities. During development of the method for this study, data were collected on reproducibility (Table S4), sample stability (Figure S1) and discriminatory power of VOC profiles from swab samples (Figure S2). Stability data on Figure S1 indicate that swab samples are unstable as chemical abundances in the headspace decrease over time. This technique is best employed for on-the-day rapid turnaround of information that could aid a diagnosis. Long- term storage of swab samples for future VOC analysis is not an option due to the lack of sorbent on swabs. However, if a GCMS is unavailable on the day of sample collection, freezing the SPME fibre post-HS sampling until analysis is a better alternative to storing the swab sample.

During an infection, pathogenic bacteria utilise a variety of specific metabolic gene pathways to out-compete resident flora and commensals for available nutrients [26]. The increased metabolic activity and cell number of pathogens in infected wounds can result in an increased abundance of volatile metabolic by-products—which, if recovered—can be used to differentiate infected wounds from non-infected wounds. A total of 42 compounds were included in this study. These compounds had widely varying prevalence across infected and non-infected wound swabs, illustrated in Figure 1. Among the most frequently detected compounds across both infected and non-infected wound swabs were acetone and ethanol. These compounds are produced by microbes through the metabolism of sugar and lipids [27]; they were also detected in relatively low abundances in blank samples and therefore have no discriminatory power. Dimethyl disulfide and dimethyl trisulfide were only detected in samples taken from severely infected wounds. Sulfur-containing volatile compounds can arise from the microbial oxidation of methanethiol [28] and can also be generated through the metabolism of the sulfur-containing amino acids, cysteine and methionine [16]—both of which are upregulated during infection. Other infrequently detected but clear discriminators of infected and non-infected samples were the primary metabolites 2-butanone, 3-methylbutanol and acetoin (Figures S5 and S6). Each of these compounds is derived from the downstream metabolism of glucose [27] and has been previously shown to be emitted by wound-associated pathogens in vitro [27,29].

VOC profiles detected in swabs taken from severely infected wounds, and wounds with osteomyelitis (Texas Score: 3B) showed higher diversity and dissimilarity from less severely infected and non-infected wounds (Figure 2). Severe wound infections have previously been associated with high species- and strain-level microbial diversity [30]. Osteomyelitis is characterised by a deep infection that penetrates tissue to the bone [1]. Among the wound VOC profiles with the highest dissimilarity was P3, a non-diabetic wound that was severely infected. The infection severity of P3 was shown by the presence of mixed anaerobes in the microbiology report (Table S1), indicating a deep chronic infection [31]. Non-infected diabetic wounds (Texas score 1A) demonstrated low volatilomic diversity and compound abundances compared to infected wounds with classifications of 1B, 2B, 3B and 1D (Figures S4–S8). Less-severely infected wounds (1B and 2B) showed low profile-level dissimilarity compared to severely infected wounds and wounds with osteomyelitis (Figure 2). The presence of SCFAs discriminated some of these wounds from non-infected wounds (Figure S4). Although there were only four patients with osteomyelitis included in this study, the discrimination of these sample VOC profiles compared to non-infected and less-severely infected patients highlights the clear potential application for VOCs osteomyelitis detection.

Strong differences between infected and non-infected groups were observed in acetic acid, butanoic acid, methylbutanoic acid, 2-methylpropanoic acid, ethyl acetate and phenylethyl alcohol (Figure 4). All 6 of these compounds have been shown to be metabolically produced by wound-associated pathogens in vitro [29,32]. During infection, microbial pathogens liberate sugars, lipids and amino acids from the biodegradation of necrotic tissue [33]. These degradation products act as the metabolic precursors for volatile SCFAs [8]. Methylbutanoic acid is produced by pathogenic bacteria as a byproduct of leucine catabolism [10]. Accumulation of SCFAs such as methylbutanoic acid contributes to the foul odour in infected wounds, especially in anaerobic infections. Emerging evidence has shown how healing and non-healing wounds are influenced by amino acid and lipid derivatives, where healing wounds show significantly higher levels of amino acids than non-healing ulcers [34]. The function behind the generation of SCFAs/BCFAs during infection is relatively unknown; however, current research suggests these molecules may act as signalling markers that regulate metabolism between microbial species and genus [35]. Although wounds 12A, 12B and 17 were infected, no SCFAs (except acetic acid) were detected from these swabs. This highlights the individuality of wound infections and validates the need for a larger-scale study.

Despite the sample size of this pilot study being relatively low, logistic regression analysis was performed on the data to test the use of specific VOCs for accurately determining the presence of infection in a larger sample cohort. ROC curves were used to visualise the individual and grouped performance of the six strong discriminator compounds for predicting infection (Figure 5). Compounds that demonstrated the highest predictive ability for infection were methylbutanoic acid, acetic acid, butanoic acid, phenylethyl alcohol, and 2-methylpropanoic acid. Methylbutanoic acid, 2-methylpropanoic acid, phenylethyl alcohol and ethyl acetate had low sensitivities (high false-negative rate); however, the specificity observed for each was 100% (ability to identify true-positives). The detection of 3-methylbutanoic acid was recently reported to be a strong predictor of *S. aureus* infection in the breath of patients with ventilator-associated pneumonia (VAP) [14]. Acetic acid was present in all samples (low specificity), but was highly abundant in infected samples (high sensitivity), which is indicative of a higher rate of pathogen primary metabolism. Pathogen-specific volatiles have not been clearly determined in this pilot study based on assessments of VOC data against the microbiology data (Table S1). However, given the complexities of polymicrobial infections, larger numbers of wounds are required to rigorously investigate this to show significance. The multiple regression model visualises the improvement in prediction performance by grouping the FA regressions to give an infection prediction accuracy of 81.6% (Figure 5). The performance of the model is dependent on a variety of factors, such as the severity of infection and microbial composition. As can be seen from Figure 2, severely infected osteomyelitis wounds (P4, P10, P15 and P19) have high dissimilarity compared to less severely and non-infected wounds. The volatile acid profile is also higher in severely infected/osteomyelitis wounds (Figure S4); therefore, the model may predict severely infected wounds more accurately than less severely infected wounds.

Following up from this work, a longitudinal study that profiles VOCs from wounds in response to wound healing or degradation from higher numbers of patients will comprehensively confirm the prevalence and potential clinical applicability of these compounds. Investigating the specific mechanisms underlying the production of infection-associated volatile metabolites will be key. Using stable isotope monitoring experiments on key wound-associated pathogens in highly specific in vitro wound models to elucidate the metabolic breakdown of labelled amino acid and carbohydrate substrates, the results of which can be validated. Such methods could also be employed to compare the metabolic mechanisms utilised by antibiotic-resistant and -sensitive pathogens under such conditions.

## 5. Conclusions

In this work, swab samples collected from infected and non-infected wounds were analysed using a rapid untargeted HS-SPME-GC-MS workflow with a turnaround time of under 3 h. Our aims were to characterise the VOC profiles of infected and non-infected wound samples; identify compound differences between the sample groups, and then to assess the performance of these discriminatory compounds for predicting infection. Swab samples collected from non-infected wounds were relatively non-diverse in their VOC make-up, whereas highly diverse VOC profiles were detected from severely infected/osteomyelitis wound swabs. VOC profiles from osteomyelitis wounds were highly dissimilar from those of less severely infected and non-infected wounds. A variety of molecular differences were observed across the infected and non-infected samples in this small sample cohort. Significant molecular differences were observed in SCFA and BCFAs (acetic acid, butanoic acid, methylbutanoic acid, 2-methylpropanoic acid) abundances between the tested infected and non-infected wound samples. However, although statistically significant differences were observed across analyses, the limited number of participants does not classify these observations as clinically significant. Although the results highlight major opportunities for further study of wound volatilomics, the limitation of low sample size cannot be understated, and future studies are required to build up knowledge in this area. The focus of our future work will be to monitor a higher number of participants and examine VOC shifts over time to characterise infection and healing cycles.

## Data Availability

Data will be made available upon request.

## Supplementary Materials

The following supporting information can be downloaded at: https://www.mdpi.com/article/10.3390/biom16050634/s1, Table S1: Clinical wound information; Table S2: University of Texas wound classification system31; Table S3: Notable compounds detected in blank swabs and indoor air samples. Only compounds that were detected in samples with peak area abundances >3 times that in blank samples were included in the study; Table S4: Reproducibility of selected skin-associated VOCs detected from triplicate swab samples from 5 participants; Figure S1: Skin swab sample (2 participants, 1 male, 1 female (n = 3)) VOC abundances after incubation times of 3, 5, 24, 48 h post sample collection; Figure S2: Hierarchical clustering of swab samples (incubation time: 2 h) collected from axilla, forearm and abdomen of a single participant during method development; Figure S3: (Left) Total ion chromatogram screenshot of wound ID 18 at 15.00–16.00 min. (Right) Mass spectrum of peak at 15.7 min subsequently identified as methylbutanoic acid (mixture of 2 isomers 2- and 3-methylbutanoic acid); Figure S4: Comparative dotplots illustrating differences in the abundance of acids across infected and non-infected wound samples; Figure S5: Comparative dotplots illustrating differences in the abundance of alcohols detected across infected and non-infected wound samples; Figure S6: Comparative dotplots illustrating differences in the abundance of ketones detected across infected and non-infected wound samples; Figure S7: Comparative dotplots illustrating differences in the abundance of hydrocarbons detected across infected and non-infected wound samples; Figure S8: Comparative dotplots illustrating differences in the abundance of various amine, amide, aldehyde, pyrrole, indole, acetate, and ester compounds detected across infected and non-infected wound samples.

## References

[1] Jeffcoate W.J., Harding K.G. (2003). Diabetic foot ulcers. Lancet.

[2] Armstrong D.G., Boulton A.J.M., Bus S.A. (2017). Diabetic Foot Ulcers and Their Recurrence. N. Engl. J. Med..

[3] Ndosi M., Wright-Hughes A., Brown S., Backhouse M., Lipsky B.A., Bhogal M., Reynolds C., Vowden P., Jude E.B., Nixon J. (2018). Prognosis of the infected diabetic foot ulcer: A 12-month prospective observational study. Diabet. Med..

[4] Salleh N.A.b.M., Tanaka Y., Sutarlie L., Su X. (2022). Detecting bacterial infections in wounds: A review of biosensors and wearable sensors in comparison with conventional laboratory methods. Analyst.

[5] Li S., Renick P., Senkowsky J., Nair A., Tang L. (2021). Diagnostics for Wound Infections. Adv. Wound Care.

[6] Lipsky B.A., Senneville É, Abbas Z.G., Aragón-Sánchez J., Diggle M., Embil J.M., Kono S., Lavery L.A., Malone M., van Asten S.A. (2020). Guidelines on the diagnosis and treatment of foot infection in persons with diabetes. Diabetes Metab. Res. Rev..

[7] Senneville É., Albalawi Z., Van Asten S.A., Abbas Z.G., Allison G., Aragón-Sánchez J., Embil J.M., Lavery L.A., Alhasan M., Oz O. (2023). IWGDF/IDSA Guidelines on the Diagnosis and Treatment of Diabetes-related Foot Infections (IWGDF/IDSA 2023). Clin. Infect. Dis. Off. Publ. Infect. Dis. Soc. Am..

[8] Azimzadeh M., Khashayar P., Mousazadeh M., Daneshpour M., Rostami M., Goodlett D.R., Manji K., Fardindoost S., Akbari M., Hoorfar M. (2025). Volatile organic compounds (VOCs) detection for the identification of bacterial infections in clinical wound samples. Talanta.

[9] Fitzgerald S., Holland L., Ahmed W., Piechulla B., Fowler S.J., Morrin A. (2024). Volatilomes of human infection. Anal. Bioanal. Chem..

[10] Weisskopf L., Schulz S., Garbeva P. (2021). Microbial volatile organic compounds in intra-kingdom and inter-kingdom interactions. Nat. Rev. Microbiol..

[11] Choong-Min R., Weisskopf L., Piechulla B. (2020). Bacterial Volatile Compounds as Mediators of Airborne Interactions.

[12] S. K., Saquib M., Poojary H., Illanad G., Valavan D., M S., Nayak R., Mazumder N., Ghosh C. (2024). Skin emitted volatiles analysis for noninvasive diagnosis: The current advances in sample preparation techniques for biomedical application. RSC Adv..

[13] Zetola N.M., Modongo C., Matsiri O., Tamuhla T., Mbongwe B., Matlhagela K., Sepako E., Catini A., Sirugo G., Martinelli E. (2017). Diagnosis of pulmonary tuberculosis and assessment of treatment response through analyses of volatile compound patterns in exhaled breath samples. J. Infect..

[14] Ahmed W.M., Fenn D., White I.R., Dixon B., Nijsen T.M.E., Knobel H.H., Brinkman P., Van Oort P.M.P., Schultz M.J., Dark P. (2022). Microbial volatiles as diagnostic biomarkers of bacterial lung infection in mechanically ventilated patients. Clin. Infect. Dis..

[15] Thomas A.N., Riazanskaia S., Cheung W., Xu Y., Goodacre R., Thomas C.L.P., Baguneid M.S., Bayat A. (2010). Novel noninvasive identification of biomarkers by analytical profiling of chronic wounds using volatile organic compounds. Wound Repair Regen..

[16] Landaud S., Helinck S., Bonnarme P. (2008). Formation of volatile sulfur compounds and metabolism of methionine and other sulfur compounds in fermented food. Appl. Microbiol. Biotechnol..

[17] Shirasu M., Nagai S., Hayashi R., Ochiai A., Touhara K. (2009). Dimethyl trisulfide as a characteristic odor associated with fungating cancer wounds. Biosci. Biotechnol. Biochem..

[18] Thuleau A., Dugay J., Dancremont C., Jemmali Z., Elard J., Ricke Y., Cassoux N., Watson S., Escande M., Fromantin I. (2018). Volatile organic compounds of malignant breast cancer wounds: Identification and odors. Wounds.

[19] Haalboom M., Gerritsen J.W., van der Palen J. (2019). Differentiation between infected and non-infected wounds using an electronic nose. Clin. Microbiol. Infect..

[20] Ratiu I.-A., Ligor T., Bocos-Bintintan V., Szeliga J., Machała K., Jackowski M., Buszewski B. (2019). GC-MS application in determination of volatile profiles emitted by infected and uninfected human tissue. J. Breath Res..

[21] Lavery L.A., Armstrong D.G., Harkless L.B. (1996). Classification of diabetic foot wounds. J. Foot Ankle Surg..

[22] Rajhathy E.M., Meer J.V., Valenzano T., Laing L.E., Woo K.Y., Beeckman D., Falk-Brynhildsen K. (2023). Wound irrigation versus swabbing technique for cleansing noninfected chronic wounds: A systematic review of differences in bleeding, pain, infection, exudate, and necrotic tissue. J. Tissue Viability.

[23] van den Berg R.A., Hoefsloot H.C., Westerhuis J.A., Smilde A.K., van der Werf M.J. (2006). Centering, scaling, and transformations: Improving the biological information content of metabolomics data. BMC Genom..

[24] Finnegan M., Fitzgerald S., Duroux R., Attia J., Markey E., O’Connor D., Morrin A. (2024). Predicting Chronological Age via the Skin Volatile Profile. J. Am. Soc. Mass Spectrom..

[25] Vogeser M., Habler K. (2026). Applications of mass spectrometry in the routine diagnostic medical laboratory—A status report 2025. J. Chromatogr. B.

[26] Rohmer L., Hocquet D., Miller S.I. (2011). Are pathogenic bacteria just looking for food? Metabolism and microbial pathogenesis. Trends Microbiol..

[27] Fitzgerald S., Holland L., Morrin A. (2021). An investigation of stability and species and strain-level specificity in bacterial volatilomes. Front. Microbiol..

[28] Schäfer H., Eyice Ö (2019). Microbial cycling of methanethiol. Curr. Issues Mol. Biol..

[29] Fitzgerald S., Furlong C., Holland L., Morrin A. (2022). Multi-strain and -species investigation of volatile metabolites emitted from planktonic and biofilm candida cultures. Metabolites.

[30] Kalan L.R., Meisel J.S., Loesche M.A., Horwinski J., Soaita I., Chen X., Uberoi A., Gardner S.E., Grice E.A. (2019). Strain and species level variation in the microbiome of diabetic wounds is associated with clinical outcomes and therapeutic efficacy. Cell Host Microbe.

[31] Coluccio A., Lopez Palomera F., Spero M.A. (2024). Anaerobic bacteria in chronic wounds: Roles in disease, infection and treatment failure. Wound Repair Regen..

[32] Bowler P.G., Duerden B.I., Armstrong D.G. (2001). Wound microbiology and associated approaches to wound management. Clin. Microbiol. Rev..

[33] King J.R., Koerber A.J., Croft J.M., Ward J.P., Williams P., Sockett R.E. (2003). Modelling host tissue degradation by extracellular bacterial pathogens. Math. Med. Biol. J. IMA.

[34] Bergner R.T., Onida S., Velineni R., Spagou K., Gohel M.S., Bouschbacher M., Bohbot S., Shalhoub J., Holmes E., Davies A.H. (2023). Metabolic Profiling Reveals Changes in Serum Predictive of Venous Ulcer Healing. Ann. Surg..

[35] McCrory C., Lenardon M., Traven A. (2024). Bacteria-derived short-chain fatty acids as potential regulators of fungal commensalism and pathogenesis. Trends Microbiol..

