# Supplementary material for: Headspace Volatile Organic Compound (VOC) Profiling of Infected and Non-Infected Wound Swabs—A Pilot Study
Source: Biomolecules. 2026 Apr 24;16(5):634. doi: 10.3390/biom16050634 (PMC13204046; doi:10.3390/biom16050634)
Supplement: Supplementary file 1 [file biomolecules-16-00634-s001.zip › biomolecules-4266499-supplementary.pdf]

Headspace Volatile Organic Compound (VOC) Profiling of Infected and Non-Infected Wound Swabs—A Pilot Study

Shane Fitzgerald [1,*], Linda Holland [2], Melissa Finnegan [1], Kellie Fortune [3], Brid Cooney [3], Eoghan O'Neill [4,5], John H. McDermott [3], Seamus Sreenan [3,6], Tommy Kyaw-Tun [3] and Aoife Morrin [1,*]

1 School of Chemical Sciences, Insight Research Ireland Centre for Data Analytics, Dublin City University, D09 V209 Dublin, Ireland; melissafinnegan33@gmail.com
2 School of Biotechnology, Dublin City University, D09 V209 Dublin, Ireland; linda.holland@dcu.ie
3 Academic Department of Endocrinology and Diabetes, Connolly Hospital, Royal College of Surgeons in Ireland, D15 X40D Dublin, Ireland; kellie.fortune@hse.ie (K.F.); brid.cooney@hse.ie (B.C.);johnmcdermott@rcsi.ie (J.H.M.); ssreenan@rcsi.ie (S.S.); tommykyawtun@rcsi.ie (T.K.-T.)
4 Department of Clinical Microbiology, RCSI University of Medicine and Health Sciences, RCSI Education &Research Centre, D09 YD60 Dublin, Ireland; eoneill@rcsi.com
5 Department of Microbiology, Connolly Hospital, D15 X40D Dublin, Ireland
6 Health Research Board Diabetes Collaborative Clinical Trial Network, H91 V4AY Galway, Ireland
* Correspondence: Correspondence: s.fitzgerald@imperial.ac.uk (S.F.); aoife.morrin@dcu.ie (A.M.)

Table S1: Clinical wound information.

**Wounds IDs 6a & 6b , 7a & 7b; and 12a & 12b were collected from different wounds on same patients.* I: infected; NI: non-infected; WBC: white blood cell count; Neut: neutrophils; CRP: C-reactive protein (CRP).

	Scan		Infection status			Bloodwork		
**Wound ID**	X-ray	MRI	Infection (I/NI)	Microbiology plating result	Texas	WCC (x10^9/L)	Neut (x10^9/L)	CRP (mg/mL)
**1**	No	No	NI	*Scanty commensals*	~~NA~~	4.2	2.4	7.5
**2**	No	No	I	*Scanty commensals*	B1	7.5	5.1	29.7
**3**	No	No	I	*Mixed anaerobes*	~~NA~~	8.2	5.7	32.1
**4**	No	No	I	*Ent. Faecalis, Acinetobacter*	B3	8.2	4.9	11.3
**5**	Yes	Yes	NI	*Mixed anaerobes, p mirabilis, Sau*	A1	7.3	4.9	3.0
**6a**	No	No	I	*P vulgarirs, ent faecalis*	B1	8.2	4.9	<0.6
**6b**	Yes	Yes	I	*P vulgarirs, BHS Gp G*	B1	8.2	4.9	<0.6
**7a**	Yes	Yes	NI	*P mirabilis*	A1	10.4	6.8	1.7
**7b**	No	No	NI	*P mirabilis*	A1	10.4	6.8	1.7

**8**	No	No	NI	*S aureus, scanty E. coli*	A1	12.2	8.3	5.1
**9**	No	No	NI	*P. mirabilis*	A1	5.5	3.2	0.9
**10**	No	No	I	*Kleb Oxytoca and Proteus Mirabilis*	B3	5.8	4	40.3
**11**	No	No	NI	*BHS Group B*	A1	6.8	4.5	2.9
**12a**	Yes	Yes	I	*Scanty S. aureus*	B2	12.7	8.7	22.9
**12b**	Yes	Yes	I	*Scanty S. aureus*	B2	12.7	8.7	22.9
**13**	No	No	NI	*Ent.faecalis , Serratia Liquef /*	A1	9.9	7.9	5.7
**14**	No	No	NI	*Scantty S. aureus, beta-haemolytic Group B Streptococcus*	A1	8.9	6.8	2.5
**15**	Yes	Yes	I	*S. aureus*	B3	12.2	9.8	249.7
**16**	No	No	NI	*P. mirabilis*	A1	7.8	5.1	4.6
**17**	Yes	No	I	*Ps.aeruginosa, Staph aureus, Ent Faecalis /*	B1	8.1	4	3.0
**18**	No	No	I	*Proteus mirabil*	B1	8.8	6	3.6
**19**	Yes	No	I	*Scanty commensals*	B3	9.4	5.3	24.0
**20**	No	No	I	*K. oxytoca, P.mirabilis, E. raffinosus*	~~NA~~			
**21**	Yes	No	I	*P. mirabilis*	D1	6.15	4.2	6.2
**22**	Yes	No	I	*BHS Group B*	D1	11.6	9.3	51.7
**23**	No	No	I	*S.aureus, F. magna*	B1	4.7	12.6	8.0

Table S2: University of Texas wound classification system[31]

		Grade			
		0	1	2	3
Stage	A	Pre- or post-ulcerative lesion completely epithelialised	Superficial wound, not involving tendon, capsule, or bone	Wound penetrating to tendon or capsule	Wound penetrating to bone or joint
	B	with infection	with infection	with infection	with infection
	C	with ischemia	with ischemia	with ischemia	with ischemia
	D	with infection and ischemia	with infection and ischemia	with infection and ischemia	with infection and ischemia

Table S3: Notable compounds detected in blank swabs and indoor air samples. Only compounds that were detected in samples with peak area abundances >3 times that in blank samples were included in the study.

**Retention time (min)**	**Compound**
5.64	Hexanal

6.751	Ethylbenzene
7.412	1-methylethyl-benzene
8.009	D-limonene
8.302	Heptanal
9.623	Styrene
10.275	Octanal
11.064	6-Methyl-5-hepten-2-one
11.936	Nonanal
13.046	2,6-Dimethyl-7-octen-2-ol
13.042	Acetic acid
13.404	Decanal
13.609	Benzaldehyde
16.727	3,4-Dimethylbenzaldehyde
17.598	5,9-Undecadien-2-one, 6,10-dimethyl-, (E)-
17.781	Benzyl alcohol
18.194	Butylated hydroxytoluene

Table S4: Reproducibility of selected skin-associated VOCs detected from triplicate swab samples from 5 participants

	**5-Hepten-2-one, 6-methyl-**	**Nonanal**	**Styrene**	**5,9-Undecadien-2-one, 6,10-dimethyl-**
**Subject**	%RSD	%RSD	%RSD	%RSD
**M1**	40	15	28	36
**M2**	12	8	52	17
**M3**	6	14	34	5
**F1**	45	6	7	51
**F2**	25	4	19	20
**Total**	26	9	28	26

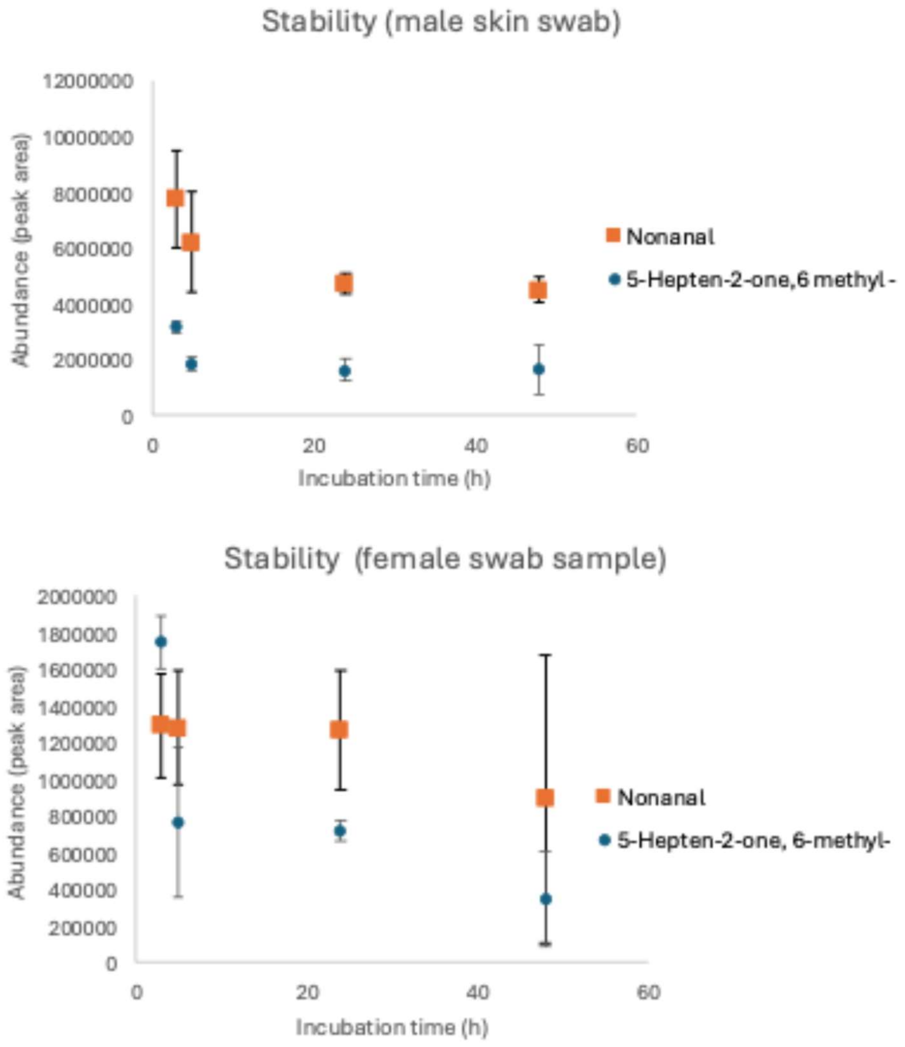

Figure S1: Skin swab sample (2 participants, 1 male, 1 female (n = 3)) VOC abundances after incubation times of 3, 5, 24, 48 h post sample collection.

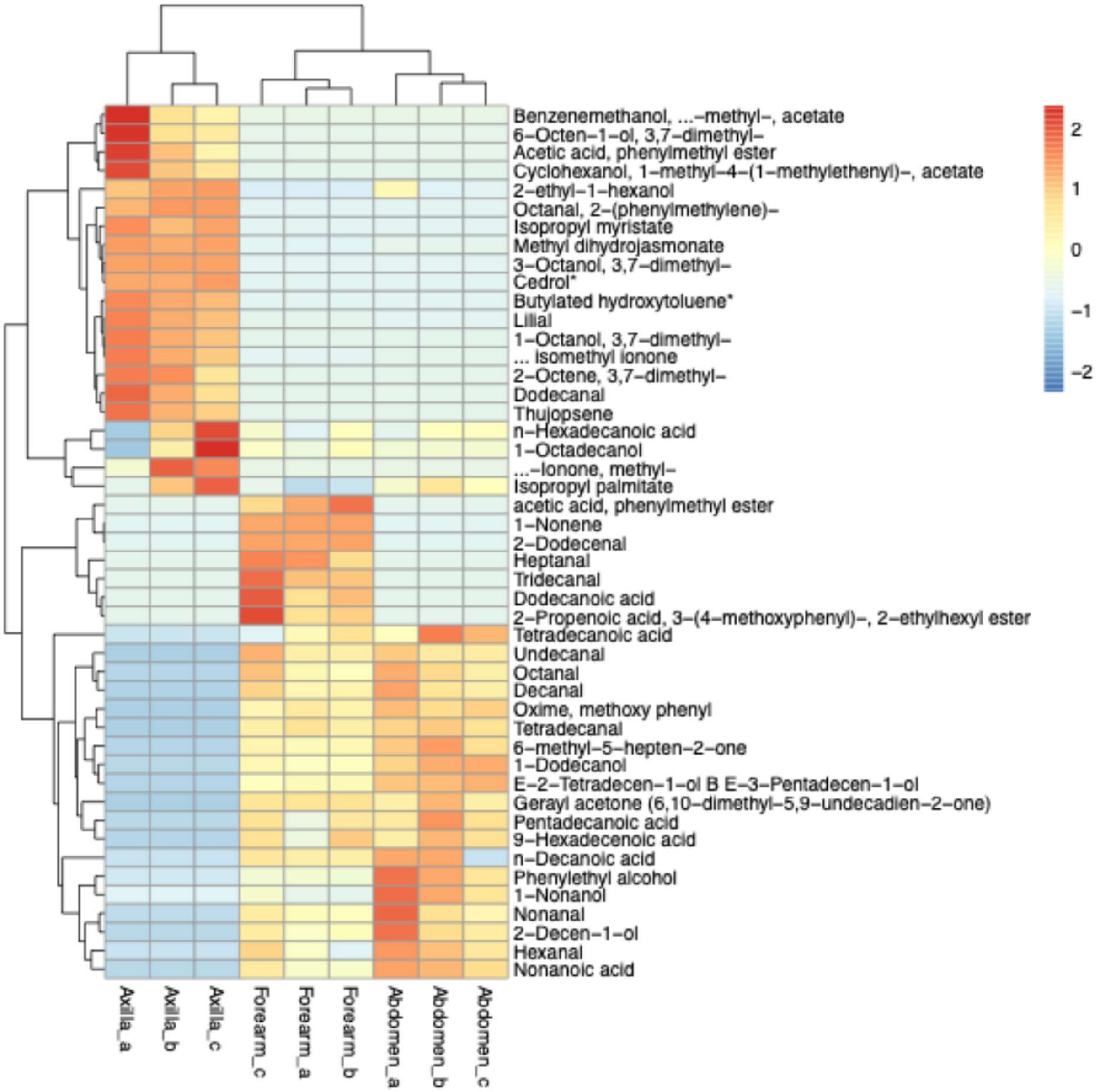

Figure S2: Hierarchical clustering of swab samples (incubation time: 2 h) collected from axilla, forearm and abdomen of a single participant during method development.

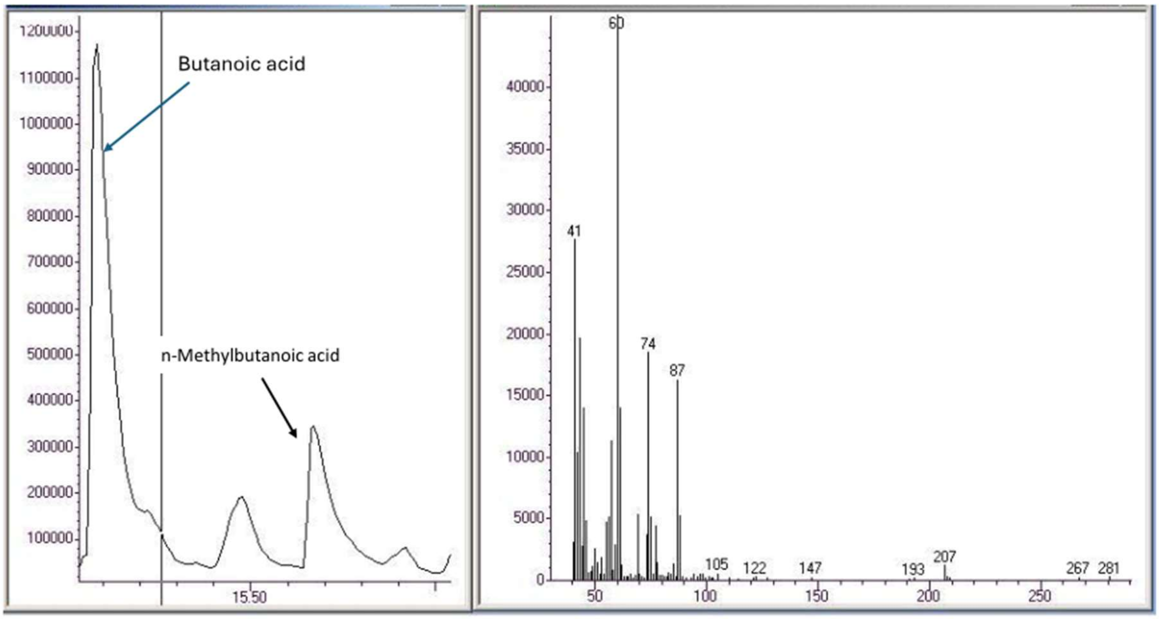

Figure S3: (Left) Total ion chromatogram screenshot of wound ID 18 at 15.00 – 16.00 min. (Right) Mass spectrum of peak at 15.7 min subsequently identified as methylbutanoic acid (mixture of 2 isomers 2- and 3-methylbutanoic acid).

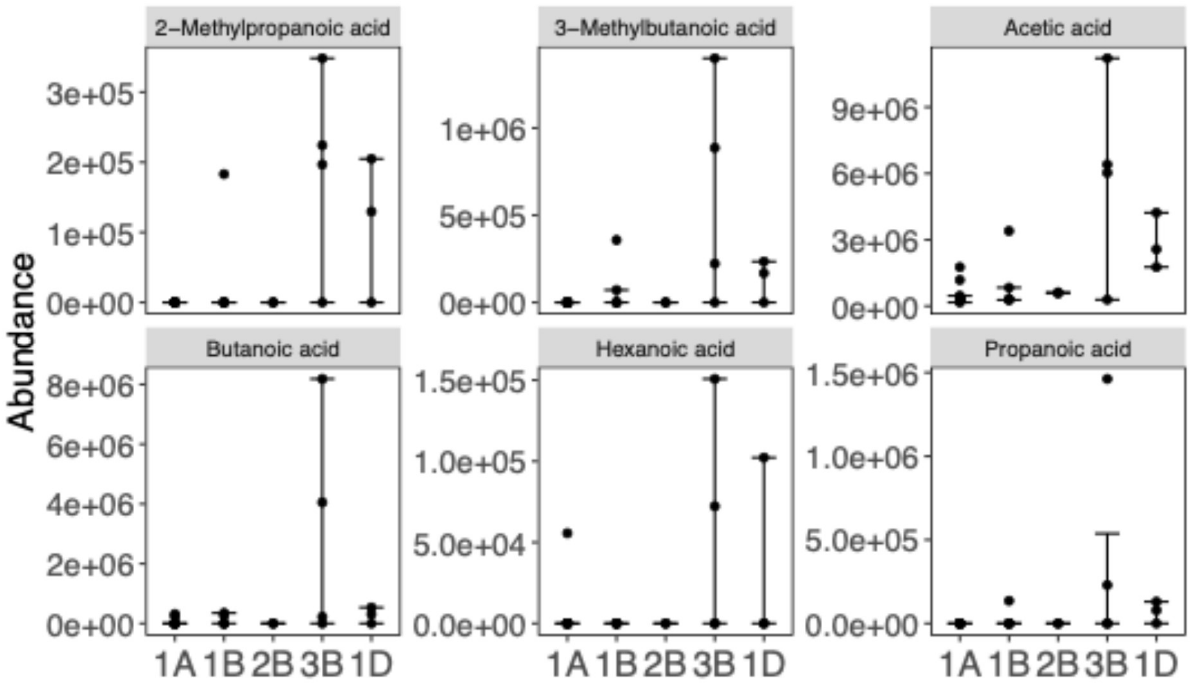

Figure S4: Comparative dotplots illustrating differences in the abundance of acids across infected and non-infected wound samples. Each plot illustrates the abundance of an individual acid recovered from DFUs classified according to Texas score. Each classification is represented: 1A (non-infected superficial wound; n= 10); 1B (infected superficial wound; n=5); 2B (infected wound penetrating tendon; n=2); 3B (infected wound penetrating to bone or joint; n=4); 1D (infected and ischemic wounds; n=2).

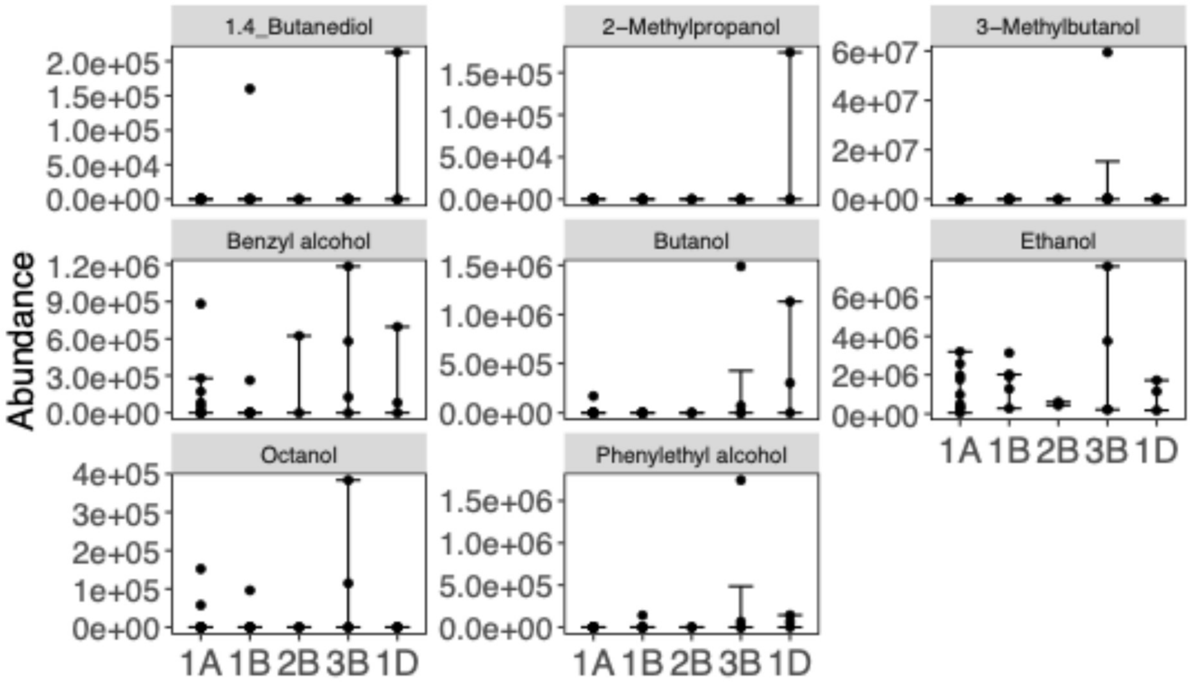

Figure S5: Comparative dotplots illustrating differences in the abundance of alcohols detected across infected and non-infected wound samples. Each plot illustrates the abundance of an individual alcohol recovered from DFUs classified according to

Texas score. Each classification is represented: 1A (non-infected superficial wound; n= 10); 1B (infected superficial wound; n=5); 2B (infected wound penetrating tendon; n=2); 3B (infected wound penetrating to bone or joint; n=4); 1D (infected and ischemic wounds; n=2).

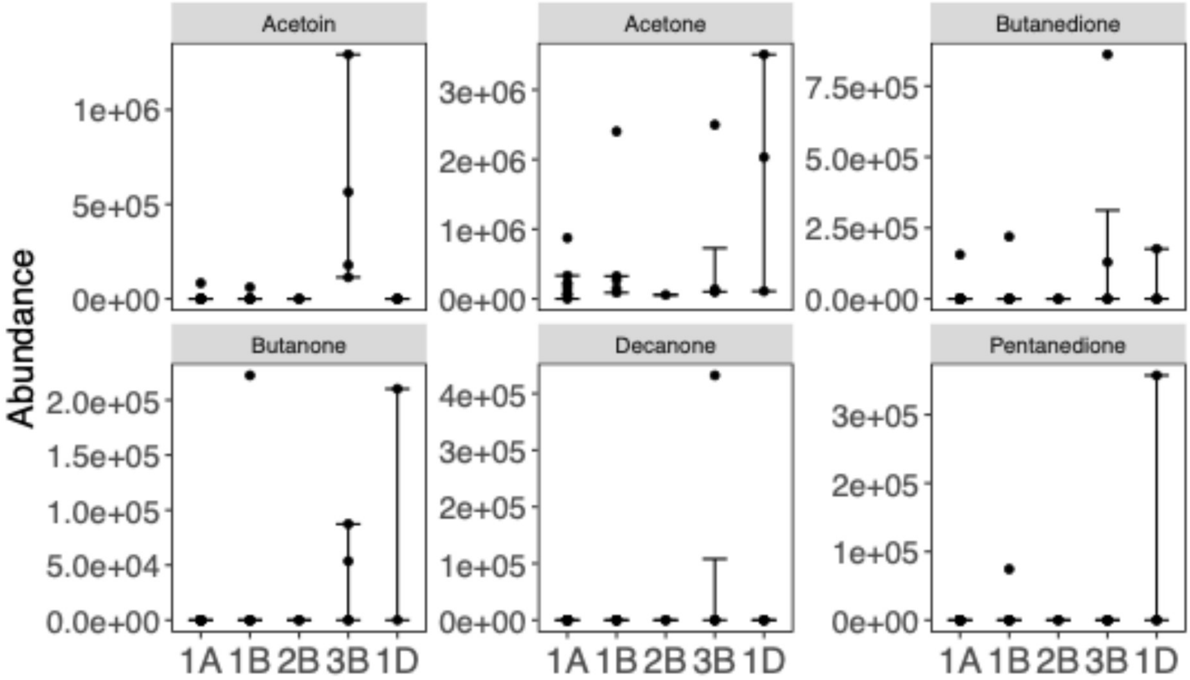

Figure S6: Comparative dotplots illustrating differences in the abundance of ketones detected across infected and non-infected wound samples. Each plot illustrates the abundance of an individual ketone recovered from DFUs classified according to Texas score. Each classification is represented: 1A (non-infected superficial wound; n= 10); 1B (infected superficial wound; n=5); 2B (infected wound penetrating tendon; n=2); 3B (infected wound penetrating to bone or joint; n=4); 1D (infected and ischemic wounds; n=2).

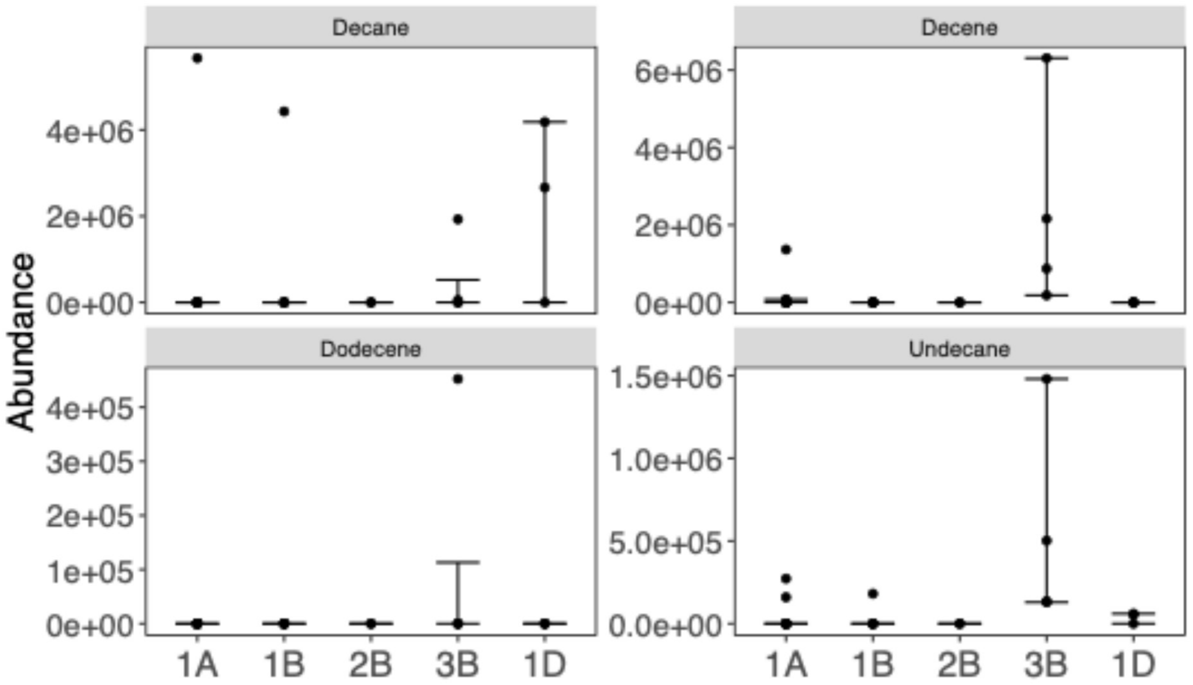

Figure S7: Comparative dotplots illustrating differences in the abundance of hydrocarbons detected across infected and non-infected wound samples. Each plot illustrates the abundance of an individual hydrocarbon recovered from DFUs classified according to Texas score. Each classification is represented: 1A (non-infected superficial wound; n= 10); 1B (infected superficial wound; n=5); 2B (infected wound penetrating tendon; n=2); 3B (infected wound penetrating to bone or joint; n=4); 1D (infected and ischemic wounds; n=2).

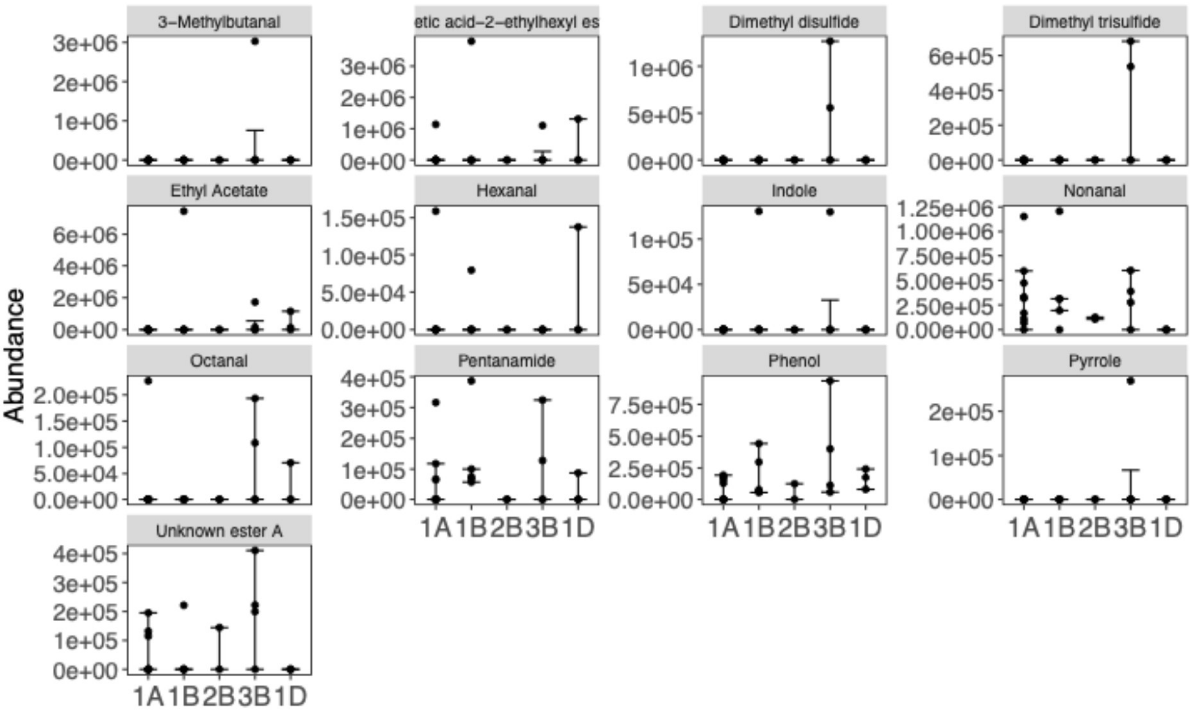

Figure S8: Comparative dotplots illustrating differences in the abundance of various amine, amide, aldehyde, pyrrole, indole, acetate, and ester compounds detected across infected and non-infected wound samples. Each plot illustrates the abundance of an individual compound recovered from DFUs classified according to Texas score. Each classification is represented: 1A (non-infected superficial wound; n= 10); 1B (infected superficial wound; n=5); 2B (infected wound penetrating tendon; n=2); 3B (infected wound penetrating to bone or joint; n=4); 1D (infected and ischemic wounds; n=2). Note: top row, center left, compound name is acetic acid-2-ethylhexyl ester.
